# Multimodal roles of transient receptor potential channel activation in inducing pathological tissue scarification

**DOI:** 10.3389/fimmu.2023.1237992

**Published:** 2023-08-29

**Authors:** Yuping Zheng, Qingrui Huang, Yanfeng Zhang, Lanxin Geng, Wuqing Wang, Huimin Zhang, Xiang He, Qiannan Li

**Affiliations:** Department of Dermatology, Shuguang Hospital Affiliated with Shanghai University of Traditional Chinese Medicine, Shanghai, China

**Keywords:** transient receptor potential channels, pathological scarification, inflammatory, fibrosis, re-epithelialization

## Abstract

Transient receptor potential (TRP) channels are a class of transmembrane proteins that can sense a variety of physical/chemical stimuli, participate in the pathological processes of various diseases and have attracted increasing attention from researchers. Recent studies have shown that some TRP channels are involved in the development of pathological scarification (PS) and directly participate in PS fibrosis and re-epithelialization or indirectly activate immune cells to release cytokines and neuropeptides, which is subdivided into immune inflammation, fibrosis, pruritus and mechanical forces increased. This review elaborates on the characteristics of TRP channels, the mechanism of PS and how TRP channels mediate the development of PS, summarizes the important role of TRP channels in the different pathogenesis of PS and proposes that therapeutic strategies targeting TRP will be important for the prevention and treatment of PS. TRP channels are expected to become new targets for PS, which will make further breakthroughs and provide potential pharmacological targets and directions for the in-depth study of PS.

## 1 Introduction

Pathological scarification (PS) is a pathological result of wound healing and is caused by inflammation and trauma. Epidemiology shows that the overall prevalence of PS is 30%-90%. The incidence of hypertrophic scar (HS) in patients with full-thickness burns is as high as 80% (1). In the United States alone, the cost of treating HS is estimated to be $400 million per year (2), which brings a huge economic burden to families and society. PS is one of the important complications of tissue damage repair, including HS and keloids. It is mainly characterized by the infiltration of inflammatory cells such as macrophages, lymphocytes, mast cells and neutrophils (3). Inflammatory mediators secreted by immune cells induce fibroblasts (FBs) fibrosis and keratinocytes (KCs) re-epithelialization, resulting in excessive deposition of a large amount of extracellular matrix (ECM) components, which is accompanied by mechanical stretching and angiogenesis and eventually develops into PS (4–6). Furthermore, recent studies have shown that oxidative stress and epigenetic regulation are involved in the occurrence of PS (7, 8). PS easily causes appearance damage and local tissue pruritus, pain, tumor-like hyperplasia or varying degrees of dysfunction, affecting the physical and mental health of patients (9). PS research has always been a challenging topic in the field of burns, plastic surgery and dermatology department (10). However, the exact pathogenesis of PS is unclear and still needs to be examined.

Recently, many studies have shown that transient receptor potential (TRP) channels are involved in many mechanisms of PS, such as participating in the development of PS fibrosis and re-epithelialization or affecting the secretion of TGF-β1 and ECM by nonimmune cells, and regulating cytokine release, cell migration and phagocytosis through immune-related mechanisms (11). Some TRP channels are involved in PS mechanical conduction, oxidative stress, epigenetics and pruritus. Therefore, the expression of TRP channels in PS deserves more attention. This review focused on specific channels in the TRP family, such as TRPV, TRPC, TRPA and TRPM, especially TRPV1-4, TRPC3, TRPC6, TRPA1 and TRPM7. These are channel proteins that play important roles in the wound healing and the development of PS.

The analysis of this information aimed to demonstrate immune inflammation and fibrosis to examine TRP channels as potential targets for inhibiting PS.

## 2 TRP channels classification and function

The TRP superfamily consists of nonselective cation channels with the ability to sense local environmental changes (12). In 1969, Cosens (13) found that light stimulation only caused a transient increase in intracellular Ca^2+^ concentrations in a Drosophila mutant with light sensing defects. Subsequently, Hardie (14) found that this was due to the lack of functional copies of genes encoding ion channels and named this type of calcium-permeable cation channel the TRP channel. According to differences in amino acid sequence homology, the mammalian TRP family is divided into six groups: ankyrin (TRPA), canonical (TRPC), melastatin (TRPM), mucolipin (TRPML), polycystin (TRPP), and vanilloid (TRPV) (15).

TRP channels are abundantly expressed in various cell types (16). For example, KCs, melanocytes, FBs and a variety of immune cells express TRP channels (17). TRP channels can be activated by external stimuli or local environmental changes (including pain, pruritus, heat, warmth or cold, odor, mechanical stimulation, and osmotic pressure changes) (18). In addition, TRP channels are critical in physiological processes such as regulating skin homeostasis, melanin synthesis, wound healing, epigenetic regulation, and pathological processes such as barrier damage, vascular stress relaxation, oxidative stress, and skin cancer caused by ultraviolet irradiation (19). There is growing evidence that the TRP family plays an important role in mediating disease fibrosis (20). These results are consistent with the mechanism of PS.

The TRPV subfamily consists of six members, which can be subdivided into heat-activated TRPV (TRPV1-4) channels and Ca^2+^-selective TRPV channels (TRPV5, TRPV6) (21), and these channels can be activated by different stimuli such as heat, pruritus, pain, osmotic pressure or chemical stimulation (21, 22). TRPV channels are involved in the activation and differentiation of immune cells and play an important role in activating macrophages, stimulating the type 17 immune inflammatory response and inducing neutrophil adhesion and chemotaxis (23–25). TRPV is closely related to fibrosis and is mainly involved in myofibroblasts (MFBs) differentiation and collagen deposition (26). TRPV is also an important osmotic-mechanical sensitive channel that mediates abnormal mechanical conduction into specific biochemical signals (23, 27). In addition, TRPV is related to angiogenesis (28), epigenetic regulation (29), electrolyte homeostasis (30) and the maintenance of barrier function (31).

The TRPC family can be further divided into four subgroups (TRPC1, TRPC2, TRPC4/5 and TRPC3/6/7) according to their amino acid sequence and functional homology (32). TRPC channels may mediate fibrotic diseases as mechanosensitive ion channels (33) and can sense and regulate oxidative stress responses. For example, the oxidation product OONO- upregulates the mRNA and protein expression of TRPC6 and TRPC3 in monocytes (34). In addition, TRPC channels are involved in the inflammatory response (35), mitochondrial metabolism in ageing (36), cell proliferation, wound healing (37), and angiogenesis (38). In particular, TRPC3 and TRPC6 have been shown to be crucial in the mechanical conduction of wound healing (39).

TRPA channels have been widely studied in the field of pruritus, pain and neurogenic inflammation (15). TRPA1 is the only member of the mammalian TRPA family that can be activated by cold and heat stimulation, mechanical forces, chemicals and endogenous signals associated with cell damage. TRPA1 is also an important mediator of acute and chronic itching perception. Exogenous and endogenous pruritus can produce scratching behavior by activating neuronal TRPA1 (19). In addition, TRPA1 is expressed in immune cells, KCs, melanocytes, FBs, epithelial cells and sensory neurons and plays a key role in the pathophysiology of almost all systems (40–42).

The TRPM subfamily consists of eight members (TRPM1-8), is the largest subfamily of TRP channels and has a specific structure and physical function (43). The TRPM subfamily is expressed in various organs and cells of the peripheral, central and immune systems and is vital in various biological processes, such as cold and heat stimulation, ion homeostasis, autophagy, vascular tension, epigenetics, and immune inflammation (29, 44–46). An increasing number of studies have shown that TRPM channel participates in fibrotic diseases (47, 48).

## 3 Cutaneous wound healing and the mechanism of PS

### 3.1 Physiological wound healing process

The physiological wound healing process is divided into four stages: hemostasis, inflammation, proliferation and remodeling (49). To a certain extent, this process is mediated by growth factors and regulatory molecules (50).

**Figure 1:**
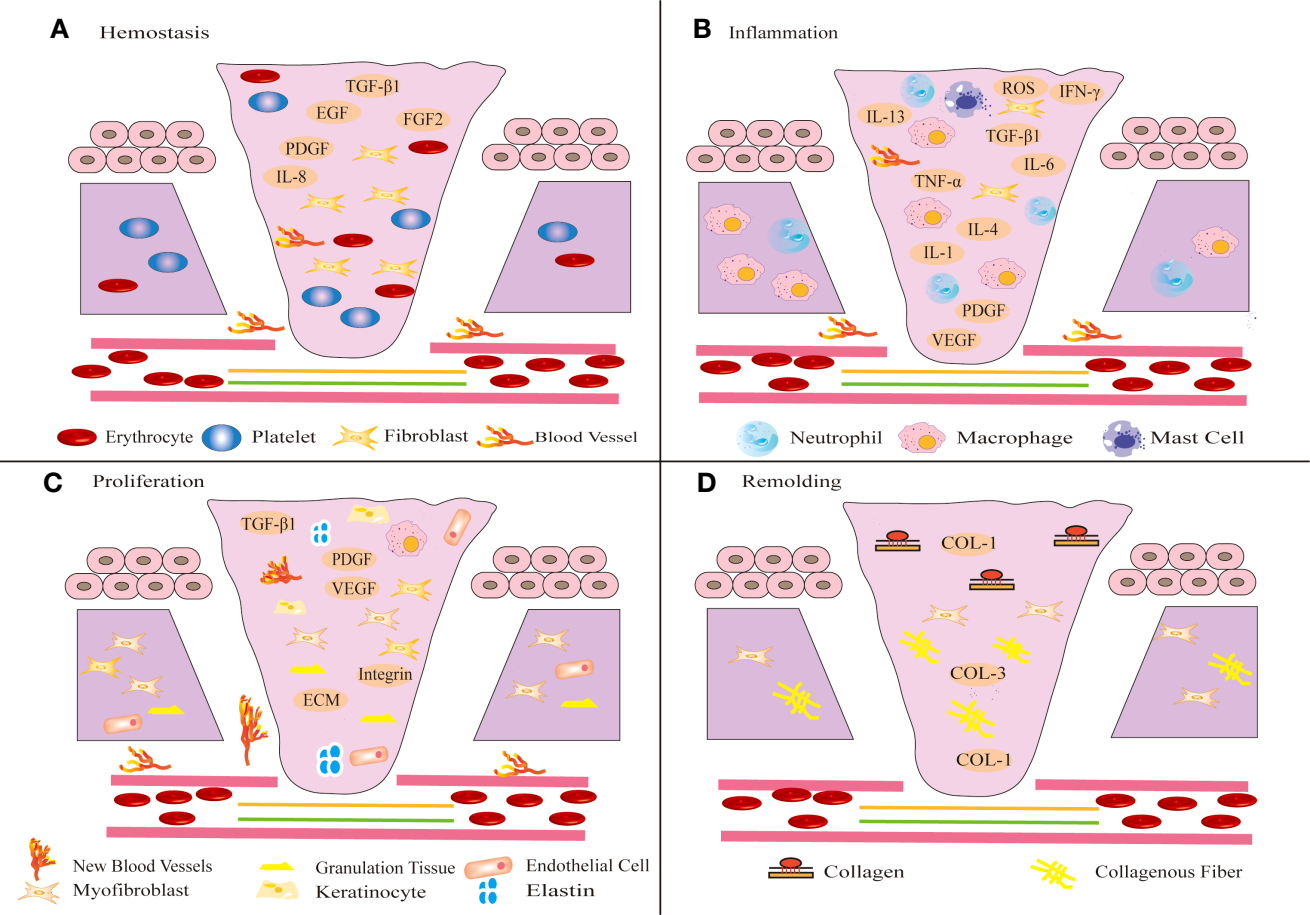
Schematic diagram of different stages of wound healing. Wound healing includes four stages: hemostasis **(A)**, inflammation **(B)**, proliferation **(C)** and remodeling **(D)**. **(A)** After injury, platelets aggregate, release chemotactic and growth factors, and eventually form clots. **(B)** Shortly thereafter, immune inflammatory cells release inflammatory mediators to resist microbial invasion and remove wound pathogens and tissue fragments. **(C)** Subsequently, FBs migrate to the wound tissue and synthesize ECM to promote the formation of granulation tissue at the wound site. The proliferation of KCs at the wound edge promotes wound re-epithelialization and migrates down to the injured dermis. **(D)** In the final remodeling stage, the tensile strength of the wound increases and the wound is completely covered by the new epidermis.

① Collagen and tissue factors promote the clumping of platelet aggregation in the affected area, releasing chemotactic and growth factors and eventually forming clots (51). ② The infiltration of inflammatory cells marks the beginning of the inflammatory phase of wound healing (50). Immune cells such as neutrophils, lymphocytes, mast cells and monocytes release inflammatory mediators to defend against microorganisms and remove wound pathogens and tissue fragments (52). When proinflammatory M1 macrophages transform into anti-inflammatory M2 macrophages, the wound healing process shifts to the proliferative phase (53). The hemostasis and inflammatory phases typically take 3 days (54). ③ The proliferative phase is an important stage associated with angiogenesis, KC migration, granulation tissue formation, ECM accumulation and epithelialization (55). FBs synthesize ECM and promote the formation of granulation tissue (53). FBs activation, KCs proliferation and migration, and new epithelial differentiation jointly promote wound re-epithelialization (56). Furthermore, FBs can be activated and differentiate into MFBs, which promote matrix remodeling to promote wound healing and angiogenesis (56, 57). ④ The final step is the remodeling phase, which typically lasts for weeks or even years (58). This stage mainly involves excessive tissue degradation (59). Excessive ECM is degraded, and collagen type III (COL-3) is replaced by mature collagen type I (COL-1), eventually leading to wound healing (60), as shown in **Figure 1**.

TRP channels play a key role in various stages of physiological wound healing, with TRPV1-4, TRPC3, TRPC6, TRPA1, and TRPM7 being closely associated. Specifically, TRPV1, TRPV3, TRPV4, and TRPA1 are involved in the inflammatory, proliferative, and remodeling phases of physiological wound healing. For instance, TRPV1 deficiency can lead to neutrophil inflammation and NETs formation, as well as defective re-epithelialization, which can prolong wound healing (61).

TRPV3, on the other hand, can induce fibrosis through TRPV3/TSLP/Smad2/3 pathway, resulting in significantly increased expression levels of α-SMA, fibronectin, COL1A1, and TSLP (62). Additionally, the selective TRPV3 activator KS0365 has been shown to accelerate the migration of KCs and promote re-epithelialization during physiological wound healing (63). Furthermore, the presence of TRPV3 in macrophage lysosomes may play a crucial role in the inflammatory phase of PS (64).

The activation of TRPV4 has been shown to promote TGF-β1 and IL-6 induced fibrosis and inflammation (20). Conversely, the lack of TRPA1 has been found to retard macrophage infiltration, subsequent fibrotic tissue formation, and mRNA expression of α-SMA and COL-1, which may further impair fibrotic behavior in fibroblasts (65). Additionally, TRPV2, TRPC3, TRPC6, and TRPM7 have been implicated in the proliferative and remodeling phases of wound healing. Specifically, TRPV2 mediates FB differentiation and contraction by promoting TGF-β1 and α-SMA expression (66).

Inhibition of TRPC3 and TRPC6 can suppress MFBs trans-differentiation and the expression of αSMA and TGF-β1 (67, 68). Furthermore, overexpression of TRPM7 promotes fibrosis and ECM deposition in wound healing (69).

### 3.2 Mechanism of PS

PS can be induced by prolonged hemostasis and inflammatory phases, leading to an abnormal increase in activated cells and their accumulation at the injury site, abnormal proliferation of FBs and excessive collagen deposition during the proliferative phase; reduced degradation of ECM and excessive wound contraction during the remodeling phase. The mechanism of PS is complex, and current research is mainly related to immune inflammation, fibrosis, re-epithelialization, epigenetics, oxidative stress, and mechanical forces.

#### 3.2.1 The mechanism by which immune cells regulate PS

**Figure 2:**
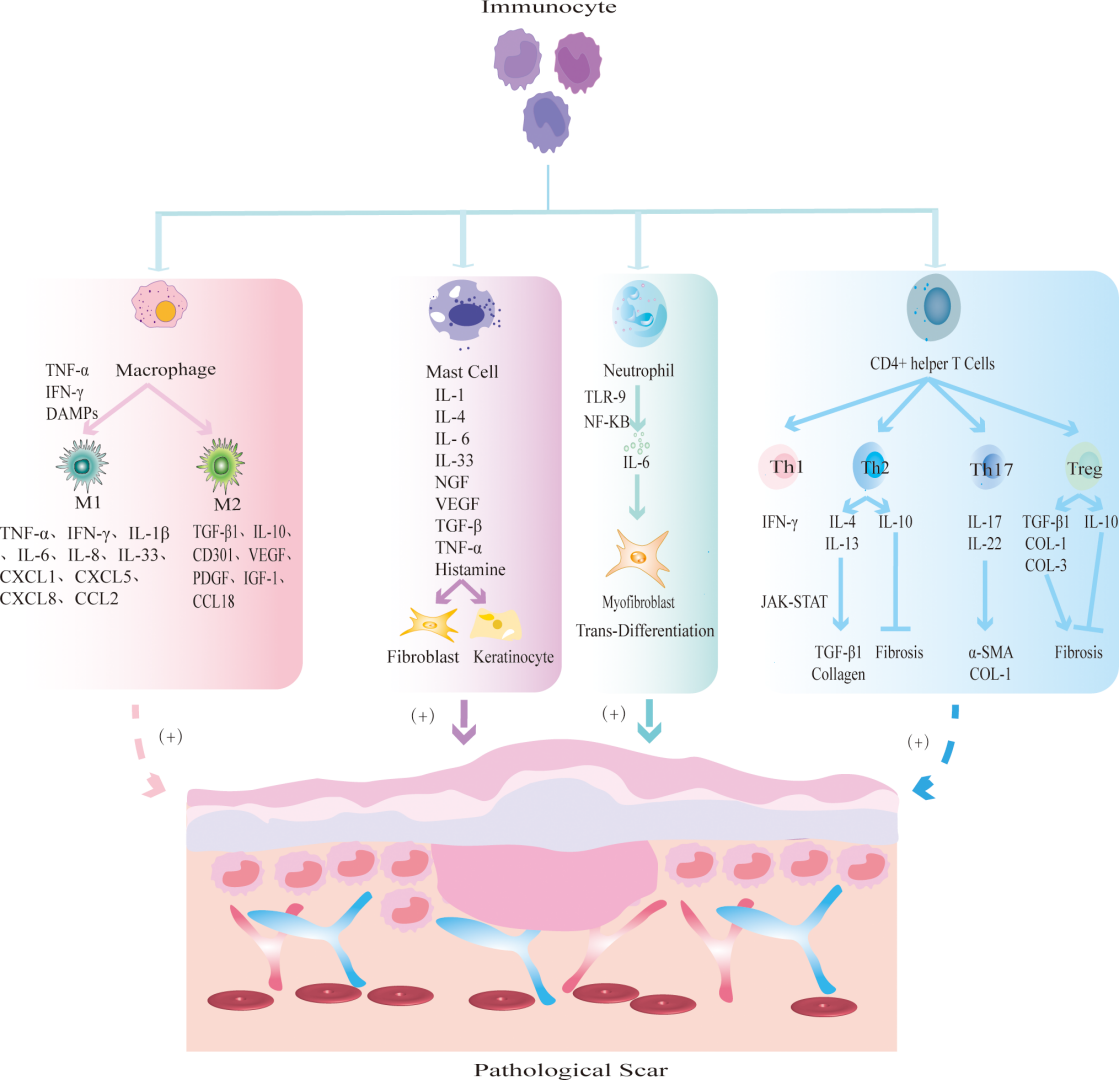
The mechanism by which different immune cells regulate PS. Macrophages are polarized into two main phenotypes: M1 and M2. M1 macrophages secrete TNF-α and IFN-γ to initiate inflammatory responses; M2 macrophages secrete TGF-β1 and IL-10 to promote tissue fibrosis. Mast cells release inflammatory mediators such as IL-1 and IL-4, and promote FBs fibrosis and KCs re-epithelialization. Neutrophils release IL-6 to induce MFBs transdifferentiation. CD4^+^ helper T cells are further divided into Th1, Th2, Th17 and Treg subsets. Th1 releases pro-inflammatory cytokine IFN-γ; Th2 cells produce anti-inflammatory factors IL-4, IL-13 to promote fibrosis and IL-10 to inhibit fibrosis; Th17 cells secrete IL-17 and IL-22 to promote the expression of α-SMA and collagen; Treg cells secrete pro-fibrotic cytokines TGF-β1, COL-1, COL-3 and anti-fibrotic cytokine IL-10.

Immune cells mainly prevent the invasion of pathogenic microorganisms during wound healing, and an imbalance will change the outcome of wound healing. Immune cells release cytokines or chemokines to promote fibrosis and re-epithelialization, resulting in excessive deposition of ECM and eventually leading to PS. At present, PS-related immune cells mainly include macrophages, lymphocytes, mast cells and neutrophils (**Figure 2**).

Macrophages are the key effector cells of innate immunity and play an important protective role in clearing pathogenic microorganisms and tissue fragments, presenting antigens, and promoting wound repair (70). Studies have shown that macrophages undergo significant phenotypic and functional changes to coordinate changes in the microenvironment at different stages of wound healing (71). In different microenvironments, macrophages can be polarized into two main phenotypes: M1 and M2. During wound healing, monocytes are polarized into the M1 phenotype by microorganisms, proinflammatory Th1 cytokines, damage-associated molecular patterns (DAMPs) and lipopolysaccharide (LPS) to initiate the inflammatory response (72, 73). Furthermore, the number of M1 macrophages begins to increase at 0-2 days after injury, peaks at 7-14 days after injury, and decreases significantly at 14-28 days after injury (72). This finding indicates that M1 cells secrete many inflammatory mediators in the early stage of normal scar formation (74, 75). During the transition from the inflammatory phase to the proliferative phase of wound healing, M1 cells are transformed into the M2 phenotype by the phagocytosis of neutrophils or the change of local wound microenvironment (76, 77). However, how M1 macrophages differentiate into M2 macrophages is not clear. The anti-inflammatory M2 phenotype is mainly involved in the proliferation and remodeling phases of wound healing. The secretion of vascular growth factors, cytokines and chemokines induces the proliferation and differentiation of FBs and MFBs, the re-epithelialization of KCs, the deposition of ECM and angiogenesis (71, 78–81). M2 cells are significantly increased at 28 days after injury and returned to baseline at 56 days (80). Normal wound healing is characterized by the transition from the early inflammatory stage, which is dominated by M1 macrophages, to the recovery stage, which is dominated by M2 macrophages (71). Increased secretion of inflammatory cytokines by M1 macrophages promotes the development of inflammation, or increased secretion of cytokines by M2 macrophages promotes the development of fibrosis, which leads to the formation of PS.

Most studies on lymphocytes in PS focus on T cells. Studies have shown that there may be a decrease in CD8^+^ cytotoxic T cells in keloids, and the number and activity of FBs co-cultured with CD8^+^ cytotoxic T cells are significantly reduced (82, 83). CD4^+^ helper T cells can be further divided into Th1, Th2, Th17 and regulatory T (Treg) cell subsets. The dynamic balance of the proinflammatory Th1 response with the anti-inflammatory Th2 response is crucial in wound healing. Once the balance is disturbed, PS may occur. During PS, Th1 cells can produce the proinflammatory factor IFN-γ to protect against fibrosis (84, 85). Th2 cells produce IL-4 and IL-13 driven by the transcription factor GATA3, which can not only induce macrophage polarization to the M2 phenotype but also induce TGF-β1 and collagen synthesis through the JAK-STAT signaling pathway and induce pruritus (86, 87). In addition, Th2 cells can produce the anti-inflammatory mediator IL-10 to protect against fibrosis (88). Treg cells can secrete cytokines and interact with other inflammatory cells to regulate PS. The profibrotic cytokines TGF-β1, COL-3, and COL-3/COL-1, anti-fibrotic cytokine IL-10 and nuclear transcription factor Foxp3 are secreted by Treg cells, which can directly regulate PS (89). Treg cells can promote macrophage polarization to the M2 phenotype and interact with helper T cells to indirectly regulate PS (88). Th17 cells activate FBs differentiation and KCs proliferation by secreting IL-17 and IL-22 (88, 90). Overall, these studies showed that lymphocytes can induce the differentiation of FBs and KCs by releasing inflammatory mediators or participate in the development of PS by interacting with macrophages. However, there are relatively few studies, and the specific mechanism needs further study.

Mast cells are mainly involved in PS and its pruritus by releasing inflammatory mediators, promoting FBs and KCs activation and excessive collagen deposition. Mast cells release inflammatory mediators, induce degranulation, directly activate FBs fibrosis, angiogenesis and KCs re-epithelialization, recruit more immune cells to migrate to the injured site; and indirectly promote tissue repair (91, 92). Furthermore, the mast cell inhibitor DSCG can reduce the width of PS and the levels of the wound inflammatory factors IL-1α, IL-1β and CXCL1 (93). In addition, mast cells are closely related to PS pruritus. Compared with those in non-pruritus keloids, the number and degranulation of mast cells in pruritus keloids were increased (94). Therefore, number of mast cells and their storage particles are important factors affecting the PS.

Neutrophils are the first immune cells to reach the wound site and secrete various cytokines to participate in wound healing. Neutrophils kill microorganisms, remove tissue debris, and contribute to the activation of macrophages (95). However, the persistent presence of neutrophils in peripheral tissues triggers an inflammatory response. Studies have shown that neutrophil extracellular traps (NETs), which are network structures by which neutrophils kill pathogens, are highly expressed in HS and induce FBs to differentiate into MFBs through the TLR-9/NF-κB/IL-6 signaling pathway (96). It is known that the IL-6 signaling pathway is critical in the pathogenesis of PS (97, 98). At present, there is a lack of studies on the specific mechanisms by which neutrophils induce FBs differentiation and interact with other immune cells to mediate PS.

#### 3.2.2 The mechanism by which nonimmune cells regulate PS

**Figure 3:**
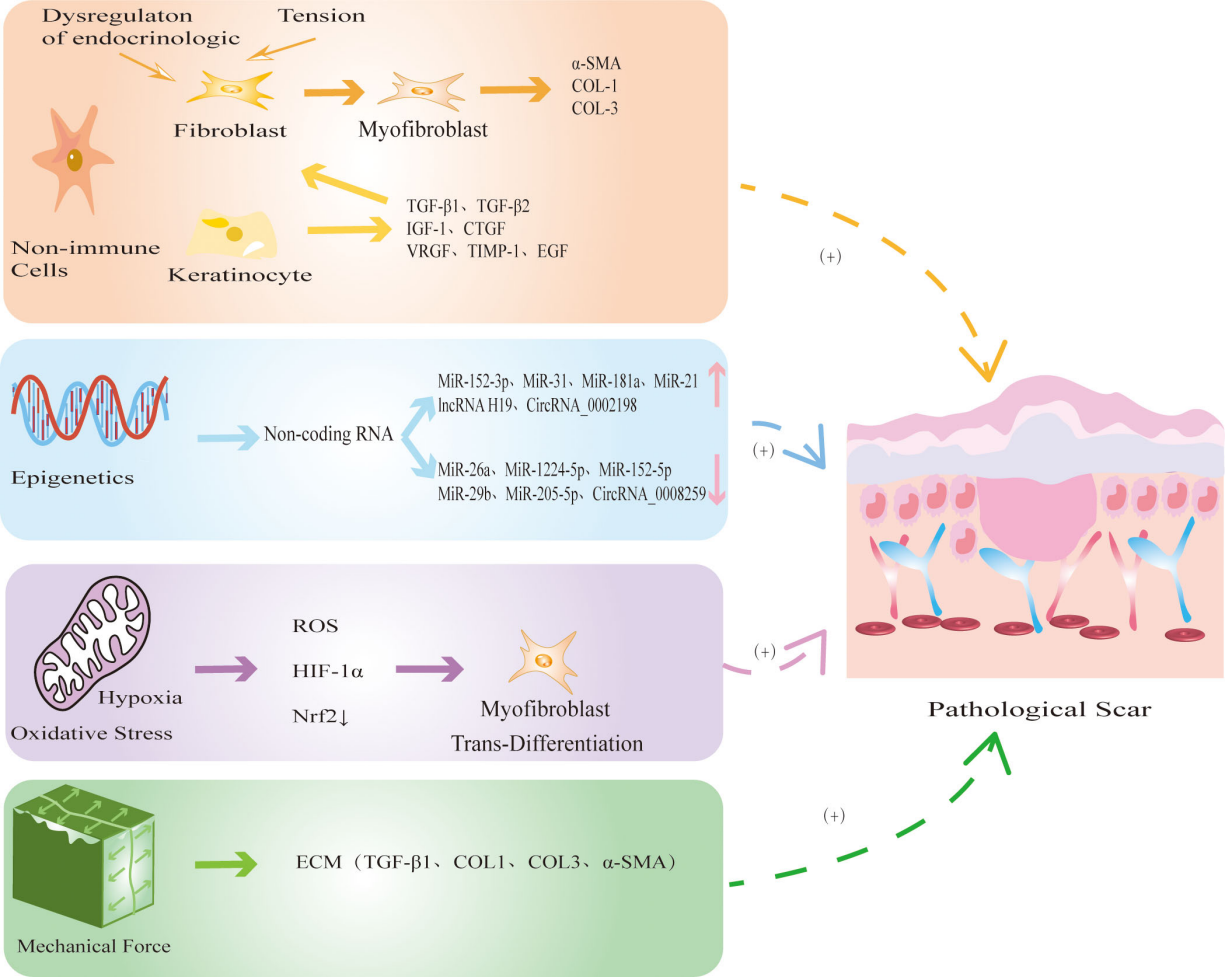
The mechanism by which non-immune cells and other factors regulate PS. Non-immune cells mainly include FBs, MFBs and KCs. When the mechanical environment around the wound changes or the internal environment is disordered, FBs differentiate into MFBs, accompanied by excessive secretion of COL-1, COL-3, α-SMA, eventually leading to PS. At the same time, KCs secretes TGF-β1 and TGF-β2 to promote re-epithelialization and FBs fibrosis. Epigenetic modification represented by noncoding RNAs is abnormally expressed in PS, which further promotes the fibrosis of FBs. Hypoxia releases ROS and HIF-1α, reduces the expression of antioxidant protein Nrf2, and induces MFBs transdifferentiation. Continuous mechanical stretching induces FBs to synthesize ECM, promotes collagen secretion and fiber synthesis.

In addition to immune cells, many nonimmune cells are involved in the development of PS (**Figure 3**). The nonimmune cells involved in PS are mainly FBs, MFBs and KCs. During physiological wound healing, MFBs undergo apoptosis or revert to static FBs. When the mechanical environment around the wound changes or the internal environment is disordered, FBs are activated by cytokines and chemokines secreted by immune cells and differentiate into MFBs, which is accompanied by excessive secretion of ECM components which ultimately leads to PS (99). MFBs mainly mediate information exchange through autocrine and paracrine mechanisms. Autocrine signaling involves binding to its own receptor to trigger TGF-β1 and induce MFBs differentiation. When TGF-β1 is inhibited, it causes the dedifferentiation of MFBs (100). In addition, the paracrine pathway mainly recruits immune cells such as macrophages and neutrophils to achieve indirect communication and jointly promote the development of PS. KCs play an important role in the development of PS by inducing wound healing re-epithelialization and regulating FBs differentiation (101). During normal skin differentiation, KCs move from the basal layer of the epidermis. When the skin is damaged, these cells proliferate and migrate to the wound, promoting wound healing (102). In HS, the thicker the epidermis, the stronger the re-epithelialization of KCs (103). In addition, KCs upregulate the expression of profibrogenic molecules to accelerate FBs proliferation and collagen production (104). At present, the abnormal interaction between KCs and MFBs is one of the most widely recognized mechanisms in PS. In conclusion, the excessive differentiation of FBs into MFBs, which in turn promotes fibrosis and excessive re-epithelialization of KCs, leads to the occurrence of PS.

#### 3.2.3 The mechanism by which other factors regulate PS

Increasing evidence shows that epigenetic modifications, represented by DNA methylation, histone modification and noncoding RNAs (ncRNAs), play a key role in the gene regulation of PS (105, 106). Many studies have focused on the expression of miRNAs in PS. The upregulation of miR-152-3p, miR-31, miR-181a, miR-21, lncRNA H19, and circRNA_0002198 (107–112) and the downregulation of miR-26a, miR-1224-5p, microRNA-152-5p, miR-29b, miR-205-5p, and circRNA_0008259 (112, 113) can promote the proliferation of FBs and the formation of collagen and ultimately induce the formation of PS (**Figure 3**).

There is hypoxia during PS. Compared with those in normal tissues, HIF-1α and ROS are highly expressed in PS (114, 115). Furthermore, hypoxia can induce the transformation of FBs to MFBs in keloids through the TGF-β1/SMAD3 pathway (114). In addition, Lee showed that antioxidant protein Nrf2 in keloids was significantly lower than that in normal skin tissue, and the protein levels of the oxidation product 2,4-dinitrophenylhydrazine were significantly higher than in normal skin (116). This finding suggests that oxidative stress is one of the mechanisms of PS (**Figure 3**).

Mechanical forces are important factors leading to PS (117, 118). TRP channels have been shown to play key roles in response to mechanical conduction, and TRPV2, TRPV4, TRPC3 and TRPC6 are potential mechanical force sensors involved in PS (3, 119). Studies have shown that continuous mechanical stretching can lead to the formation of PS by inducing FBs to synthesize ECM, indirectly activating the immune response to promote collagen secretion and fiber synthesis (120). In addition, local high mechanical forces are associated with abnormal skin fibrosis (1). These studies showed that mechanical forces can not only induce the release of inflammatory mediators by activating the immune response but also directly promote fibrosis in PS (**Figure 3**).

## 4 Modulation of TRP channels in PS

TRP channels are involved in PS (**Table 1**). The specific manifestations include immune inflammation, fibrosis, re-epithelialization, abnormal oxidative stress, epigenetic disorders, and excessive mechanical stretching. The factors are related to TRP channels. In addition, some TRP channels mediate PS-induced pruritus. TRP channels that are most closely related to PS are described below. Furthermore, we offer some opinions for reference in expounding the related problems.

**Table 1:** The function of TRP channels in PS.

Subfamilies	Positive biomarkers	Role of TRPs in PS	Influence mechanism	References
TRPV1	IL-1	Epidermal barrier disruption up-regulates the expression of TRPV1, promotes KCs release of IL-1, TNF-α, IL-6, IL-8 and GM-CSF to induce proliferation and re-epithelialization	Re-epithelialization	(121)
	TNF-α			
	IL-6			
	IL-8			
	GM-CSF			
	CGRP	TRPV1 activates the release of CGRP, promotes the expression of COL-1, TGF-β1 and α-SMA, upregulates the levels of macrophage-related inflammatory factors IL-1, IL-6, TNF-α and CCL2 through NF-κB and ERK signaling pathways, and promotes the release of IL-17 from type 17 inflammation	Immune inflammation, Fibrosis	(25, 122)
	SP	SP released by TRPV1 binds to its receptor NK1R to mediate neurogenic pruritus or activate Th2 immune cells, promoting the release of IL-4 and IL-13 to mediate pruritus	Pruritus	(87, 123–126)
	IL-31	The activation of TRPV1 promotes mast cells to release IL-31 and FBs to produce Periostin to induce pruritus		(127, 128)
	Periostin			(129–131)
TRPV2	TGF-β1	The activation of TRPV2 promotes the expression of TGF-β1 and α-SMA	Re-epithelialization	(132–134)
	α-SMA		Fibrosis	
TRPV3	COL-1	The activation of TRPV3 promotes the expression of COL-1, TGF-β1, α-SMA and fibronectin in FBs through Smad2/3 signaling pathway	Fibrosis	(62)
	TGF-β1			
	α-SMA			
	Fibronectin			
	NO	TRPV3 promotes the expression of COL-1 by activating iNOS to induce NO synthesis	Re-epithelialization	(135, 136)
	TGF-α	TRPV3 channel promotes KCs to release TGF-α, and induces KCs proliferation through TGF-α/EGFR signaling pathway		(137)
	TSLP	The upregulation of TRPV3 channel increases the expression of TSLP and PAR2 to induce pruritus	Pruritus	(138–140)
	PAR2			
TRPV4	IL-6	The activation of TRPV4 channel may promote FBs differentiation into MFBs by upregulating IL-6	Fibrosis	(20)
	TSLP	TRPV4 induces KCs to release TSLP to promote pruritus	Pruritus	(141, 142)
	Piezo1	TRPV4 cooperates with Piezo1 channel to promote mechanical conduction	Mechanical forces	(118, 119)
TRPC3	COL-1	Repeated mechanical stretching activates TRPC3 channel and promotes COL-1, TGF-β1, α-SMA and fibronectin through the Smad3/NF-κB signaling pathway	Mechanical forces	(33)
	TGF-β1			
	α-SMA			
	Fibronectin			
	NFAT	Repeated mechanical stretching induces ET-1 in KCs to bind to EDNRB in FBs to promote the expression of TRPC3 and the profibrotic gene NFAT through Gαq-PLC-DAG signaling.	Fibrosis	(143)
	ROS	The activation of TRPC3 evokes ROS release to participate in FBs differentiation	Oxidative stress	(67)
TRPC6	NFAT	TGF-β1 up-regulates the expression of TRPC6 to activate NFAT through p38 MAPK/SRF pathway, promotes MFBs transdifferentiation and the release of α-SMA and collagen	Fibrosis	(68, 144)
TRPA1	IL-4	IL-4 and IL-13 activate TRPA1 to mediate PS fibrosis through TGF-β/SMAD and IL-4Rα/STAT6 signaling pathways; IL-4 and IL-13 stimulate TRPA1 neuronal expression and induces pruritus	Fibrosis, Pruritus	(87)
	IL-13			
	IL-17	TRPA1 activates type 17 immune cells to release IL-17, IL-22 and further recruits γδT cells to release Fgf9	Immune inflammation	(92, 145, 146)
	IL-22			
	COL-1	The activation of TRPA1 promotes COL-1 through TGF-β1	Fibrosis	(147)
	TSLP	TRPA1 promotes the expression of TSLP		(126, 147)
	Periostin	The activation of TRPA1 promotes FBs to produce Periostin to induce pruritus	Pruritus	(129–131)
TRPM7	COL-1	The activation of TRPM7 enhances COL-1, TGF-β1 and α-SMA through PI3K-AKT signaling pathway	Fibrosis	(145)
	TGF-β1			
	α-SMA			
	HIF-1α	Hypoxia upregulates TRPM7 to activate STAT3/SMAD3/HIF-1α signaling pathway to induce fibrosis	Oxidative stress	(146)

### 4.1 TRPV1

**Figure 4:**
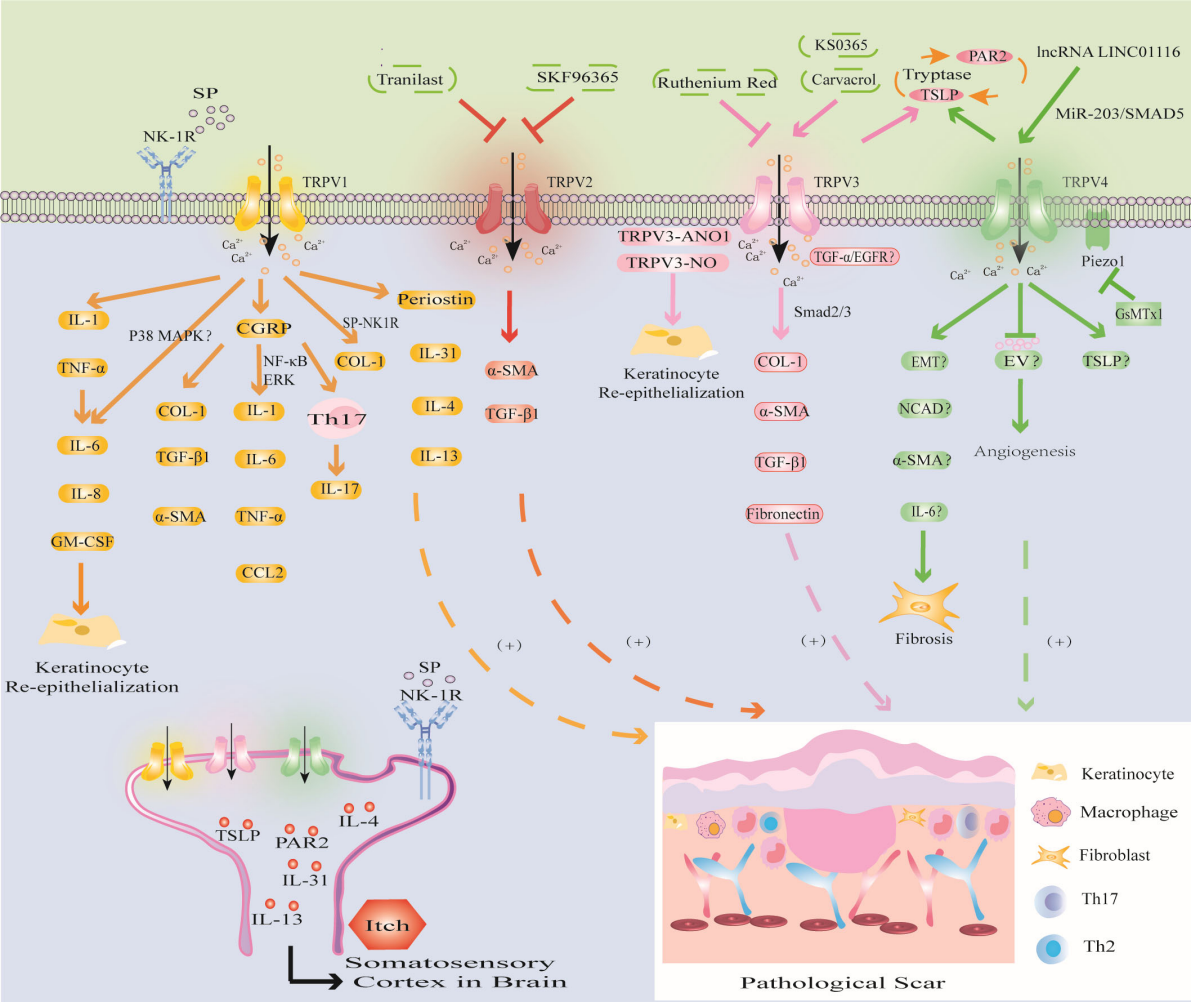
Modulation of TRPV channels in PS. TRPV1 channel activates IL-1 and TNF-α, triggers inflammatory response, promotes KCs proliferation and re-epithelialization; CGRP released by TRPV1 directly promotes PS fibrosis, and can induce PS by upregulating the levels of inflammatory factors through NF-κB and ERK signaling pathways; the SP released by TRPV1 activation mediates PS fibrosis and pruritus. The activation of TRPV1 promotes the expression of Periostin, IL-4, IL-13 and induces PS pruritus. (2) TRPV2 inhibitors SKF96365 and tranilast inhibited PS by downregulating TGF-β1 and α-SMA. (3) TRPV3 activator carvacrol promotes fibrosis through Smad2/3 pathway; activation of TRPV3 may interact with ANO1 and NO or promote PS through TGF-α/EGFR signaling pathway. PS pruritus may be related to the PAR2-TSLP positive feedback pathway mediated by TRPV3 and TRPV4 channels. (4) The activation of TRPV4 may promote the expression of EMT, NCAD, α-SMA, IL-6 to induce fibrosis, inhibit EV to inhibit abnormal angiogenesis, or release TSLP to induce PS pruritus and cooperate with Piezo1 channel to promote the formation of PS induced by mechanical stretching.

TRPV1 is not only a heat-activated capsaicin receptor but also a multimodal receptor that is activated through multiple pathways in different microenvironments and mediates PS fibrosis, re-epithelialization and inflammatory responses, as well as being involved in PS pruritic signaling (**Figure 4**) (26, 148, 149).

In HS, the expression of TRPV1 is upregulated. Studies have shown that trans-epidermal water loss (TEWL) in HS is significantly higher than that in normal skin (150). This finding suggests that there is the presence of epidermal barrier dysfunction in HS, which is consistent with the clinical manifestations of skin dryness in some HS patients. Studies have shown that the barrier function of the skin is closely related to the expression of TRPV1 in KCs. Overactivation of TRPV1 delays the recovery of epidermal barrier function (151). Further studies have shown that disrupting the water barrier leads to KCs production of IL-1 and TNF-α to trigger the inflammatory response, and upregulate IL-6, IL-8, and granulocyte/macrophage colony stimulating factor (GM-CSF), promote KCs proliferation and re-epithelialization, and ultimately induce PS (121). In addition, Capsaicin induces increases in proliferation through IL-6 upregulation and TRPV1 induces the proliferation of human corneal epithelial cells through global MAPK activation (152, 153). These studies have shown that the expression of TRPV1 is related to the barrier function and inflammatory response of HS. The loss of TRPV1 inhibited inflammatory cell invasion and expression of TGF-β1 and other proinflammatory gene expression in cultured ocular fibroblasts (154). This is due to the overexpression of TRPV1 in HS which affects KCs proliferation and differentiation (20). Therefore, interfering with TRPV1 expression in KCs may become a new therapeutic strategy for PS. In addition, studies have shown that TRPV1 activation can release calcitonin gene-related peptide (CGRP) stored in vesicles from nerve endings to mediate immune inflammation and local vasodilation (155, 156). Zhou found increased CGRP levels in both human and mouse HS tissues. Furthermore, CGRP antagonists can directly reduce the expression of COL-1, TGF-β1 and α-SMA and can downregulate the levels of the macrophage-related inflammatory factors IL-1, IL-6, TNF-α and CCL2 through the NF-κB and ERK signaling pathways. Moreover, CGRP can promote PS by inducing the Th17 immune response (122). Cohen found that IL-17 expression was closely related to the Th17 immune response in light-simulated TRPV1-Ai32 mice (25). However, this process requires TRPV1 to induce CGRP release (25). The development of macrophages, the Th17 inflammatory response and fibrosis in HS induced by CGRP may be related to the release of TRPV1 from neurons. The neuroimmune mechanism by which TRPV1 regulates PS provides a new research direction. In summary, these studies have shown that TRPV1 may promote PS by directly promoting fibrosis and re-epithelialization and indirectly inducing inflammatory stimulation.

Pruritus is the most important symptom affecting the quality of life of PS patients. TRPV1 inhibitors may be one of the effective treatment strategies. The study found that the degree of scar pruritus after TRPV1 gene knockout was significantly less than that of wild type rats (152). Further studies have shown that TRPV1 can induce pruritus by promoting the expression of the pruritus mediators IL-31 and SP (127). The level of IL-31 secreted by mast cells was increased in HS compared with that in normal tissues, and the number of mast cells was also increased (128). This finding suggests that HS pruritus may be related to TRPV1-mediated promotion of mast cell degranulation and the release of inflammatory factors. In addition, the expression of SP and TRPV1 were significantly higher in HS skin than in normal skin. Furthermore, immunofluorescence analysis showed that the distribution of TRPV1 and SP was consistent (157). As a neuropeptide, SP mediates angiogenesis, macrophage polarization, mast cell degranulation, KCs proliferation and fibrosis and is an important neuromodulator of pruritus. SP can selectively bind to its specific receptor neurokinin-1 receptor (NK-1R) to mediate neurogenic pruritus (123, 125). Further studies have shown that the SP-NK1R signaling pathway promotes FBs to secrete COL-1 and is positively correlated with SP levels (157). The mechanism of skin neurogenic pruritus mediated by the TRPV1-mediated SP-NK1R signaling pathway may provide a new therapeutic target for PS pruritus conduction. Study has confirmed that hyperbaric oxygen therapy (HBOT) can alleviate the pruritus symptoms of keloid patients by reducing the expression of TRPV1 (158), but the specific mechanism is still unclear. Recently, Hashimoto found that the new pruritic Periostin was upregulated in PS (129). Periostin is produced by TGF-β1 and histamine-stimulated FBs and induces pruritus by binding to the aVb3 integrin receptor or inducing the Th2 cytokine cascade (130). However, this process requires the activation of TRPV1 and TRPA1 (130, 131). The discovery of Hashimoto provided a new direction for researching the mechanism of Periostin-mediated pruritus in PS through the activation of TRPV1 and TRPA1. Overall, these results indicate that targeting TRPV1 channels may be a prospective therapeutic strategy for PS.

### 4.2 TRPV2

TRPV2 is mainly involved in PS by promoting the release of TGF-β1 from KCs and the differentiation of FBs (**Figure 4**).

In new granulation tissue in the wound, KCs, FBs and macrophages at the edge of the wound promote wound re-epithelialization by secreting cytokines such as TGF-β1 and stimulate MFBs to produce α-SMA, eventually leading to PS (132, 133). In the cell collagen contraction model based on this theory, researchers found that the TRPV2 inhibitor SKF96365 or tranilast inhibited collagen contraction, while a TRPV2 inhibitor or TRPV2 knockdown using siRNA reduced TRPV2 agonist-induced Ca^2+^ influx in FBs. It was further confirmed that the TRPV2 inhibitors SKF96365 and tranilast could induce FBs differentiation and collagen contraction by downregulating TGF-β1 and α-SMA expression (134). These studies showed that altering FBs and KCs differentiation with drugs targeting TRPV2 channels is beneficial for preventing PS and contracture.

### 4.3 TRPV3

TRPV3 plays an important role in mediating KCs re-epithelialization and FBs fibrosis and promoting ECM deposition in PS. In addition, TRPV3-mediated PS pruritus may be related to the expression of PAR2 and TSLP (**Figure 4**).

Studies have shown that the new TRPV3 channel activator KS0365 promotes wound healing by accelerating the re-epithelialization of KCs, while the broad-spectrum channel blocker ruthenium red and siRNA-mediated TRPV3 knockdown inhibit this process (63, 159). This finding indicates that overexpression of TRPV3 channels can promote excessive wound healing leading to the formation of PS. A clinical study showed that the TRPV3 activator carvacrol could promote the expression of COL-1, α-SMA, TGF-β1 and fibronectin in FBs through the Smad2/3 pathway, thereby promoting HS fibrosis (62). In addition, NO is an important mediator involved in biological processes such as wound healing, fibrosis, inflammation and KCs differentiation (160). Cobbold found that compared with those in normal tissues, NO levels in keloids were increased, and NO produced by nitric oxide synthase (iNOS) promoted the expression of COL-1 (135). TRPV3 induces NO synthesis by activating iNOS, thereby promoting KCs re-epithelialization and facilitating wound repair (136). This finding suggests that TRPV3-induced NO overexpression leads to PS. In addition, epidermal growth factor receptor (EGFR) can promote wound healing by promoting KCs proliferation, inflammation and angiogenesis (161). Aijima found that the phosphorylation of EGFR in the oral epithelial cells of TRPV3-KO mice was inhibited, and TGF-α, which is a ligand of EGFR, was released from KCs through the activation of TRPV3 (137). This finding indicates that the TGF-α/EGFR signaling pathway plays a role in oral mucosal wound healing through TRPV3. Although the oral mucosa repairs faster than skin wounds and has fewer scars (137), the mechanism of oral mucosal repair suggests that the activation of TRPV3 may promote PS through the TGF-α/EGFR signaling pathway. Recent studies have shown that anoctamin1 (ANO1), a calcium-activated chloride channel, can promote the migration and proliferation of cancer cells (162). The interaction of TRPV3 with ANO1 promotes the proliferation of KCs during wound healing, while TRPV3 and ANO1 inhibitors inhibit the proliferation of KCs (162). The mechanism of the TRPV3-ANO1 interaction may provide a new target for PS.

PS pruritus may be related to the expression of PAR2 and TSLP, which is mediated by TRPV3 channels. The expression of PAR2 can be detected in burn scars with pruritus (138). Furthermore, inhibiting TRPV3 channels can reduce the expression of PAR2 and inhibit the itching of burn scars (139). In addition, compared with normal tissues, the expression of TRPV3 and TSLP in KCs in burn scars was upregulated, especially in burn scar tissues with pruritus (139). Further studies have shown that the synergistic effect of TSLP and PAR2 is particularly important in mediating pruritus signal transduction. TSLP triggers mast cell degranulation and the release of tryptase by binding to its receptor. Tryptase binds to PAR2 in KCs and activates TRPV3 channels to induce Ca^2+^ influx to promote the expression of TSLP, forming a positive feedback loop. TSLP binds to its receptor and transmits to the spinal dorsal root ganglion to induce pruritus (140). Kim found that higher levels of the TRPV3 activator carvacrol were associated with higher NRS scores of the burn scar pruritus index (138). Therefore, the expression of TRPV3 in PS may be positively correlated with the degree of pruritus, and the upregulation of TRPV3 channels may be related to the increased expression of PAR2 and TSLP and the involvement of the PAR2-TSLP positive feedback pathway. The current research results provide a reference for the function of TRPV3 in PS pruritus. Further research is expected to determine whether the combination of TRPV3 with TSLP and PAR2 inhibitors can provide a feasible solution for PS pruritus.

### 4.4 TRPV4

Recent literature shows that TRPV4 may be involved in PS fibrosis, angiogenesis, pruritus, mechanical conduction and epigenetic regulation (**Figure 4**).

Epithelial-mesenchymal transition (EMT) plays an important role in wound healing by inducing re-epithelialization and promoting MFBs contraction and the secretion of ECM (163, 164). Sharma found that TGF-β1-induced EMT-like changes in KCs were dependent on TRPV4. Furthermore, TRPV4 promoted the expression of the mesenchymal markers N-cadherin (NCAD) and α-SMA in a bleomycin-induced mouse skin fibrosis model (165). Whether the activation of TRPV4 is involved in EMT in PS and affects the levels of NCAD and α-SMA remains to be further studied. In addition, studies have shown that IL-6 deficiency in TRPV4-deficient corneal FBs decreases MFBs differentiation, resulting in delayed corneal wound closure (20). It is well known that the IL-6 signaling pathway plays an important role in the pathogenesis of PS (97, 98). However, in the alkali burn wound healing response of TRPV4-null mice, biomarker gene expression of fibrosis, collagen1a1 and α-SMA were attenuated along with macrophage release of IL-6 whereas TGF-β release was unchanged (166). Therefore, TRPV4 channel may promote the differentiation of FBs into MFBs to mediate the development of PS, but whether the release of IL-6 promotes this process needs further study. Recent studies have shown that extracellular vesicles (EVs) are involved in skin wound healing (167). *Lactobacillus delbrueckii*-derived EVs (LDEVs) may inhibit PS fibrosis by inhibiting the expression of collagen and α-SMA (168). Furthermore, Wnt4 in mesenchymal stromal cell-derived extracellular vesicles (MSC-EVs) stimulated the proliferation and migration of FBs and KCs in a dose-dependent manner, enhanced the production of collagen and fibronectin to accelerate the process of wound healing (169, 170). In addition, studies have shown that EV induces abnormal angiogenesis by downregulating TRPV4-mediated ERK phosphorylation and activating VEGFR2 and YAP signaling (171). This finding suggests that studying the activity and participation of EVs in PS will provide new intervention targets for exploring the mechanism of TRPV4-mediated PS. In summary, these studies provide new ideas for TRPV4-induced PS fibrosis and angiogenesis, and the related mechanisms need to be further elucidated.

TRPV4 may also be involved in PS pruritus. Yang found that TRPV4 mRNA expression was significantly increased in patients with burn scar pruritus and was positively correlated with pruritus intensity compared with that in patients without scar pruritus (141). Lee found that skin dryness relied on TRPV4 channels to induce TSLP production in KCs and promote pruritus (142). This study provides a new research direction for the mechanism of TRPV4-mediated PS pruritus.

In addition, TRPV4 is related to the mechanical conduction of PS, and immune inflammatory cells and FBs fibrosis are involved. Studies have revealed the effect of TRPV4 on the reaction of implanted foreign bodies, and in the absence of TRPV4, macrophage-induced FBs differentiation into MFBs was significantly reduced (172). This finding suggests that TRPV4-mediated mechanical conduction contributes to the accumulation of MFBs. In addition, studies have shown that TRPV4 activation by some stresses (excessive mechanical, osmotic, and chemical stimulation) induces pain through ATP release in human corneal epithelial cells (173). Therefore, the interaction between mechanical conduction, immune cells and fibrosis may be related to TRPV4. In addition, a novel mechanically activated cation channel Piezo1 is overexpressed in HS, and its inhibitor GsMTx1 can protect rats from stretch-induced HS (118). Furthermore, Piezo1 interacts with TRPV4 after activation to produce a continuous Ca^2+^ signal and promote mechanical conduction (119). Therefore, TRPV4 can promote PS by cooperating with the Piezo1 channel, and Piezo1 blockers may be used to treat PS. These findings provide new research targets for reducing PS mechanical contraction.

Furthermore, TRPV4 may be involved in the epigenetic regulation of PS. In liver fibrosis, TRPV4 is a direct target of miR-203 and promotes TGF-β1-induced hepatic stellate cell proliferation (174). Studies have shown that miR-203 regulates wound healing and scar formation by inhibiting Hes1 expression in epidermal stem cells (175). Furthermore, studies have shown that downregulating lncRNA LINC01116 inhibits keloid by regulating the miR-203/SMAD5 axis. Western blot analysis showed that lncRNA LINC01116 and SMAD5 were upregulated in keloids, while miR-203 expression was downregulated (176). Therefore, downregulating miR-203 expression and upregulating lncRNA LINC01116 expression may promote PS by regulating TRPV4 channel, but the specific mechanism needs further research.

### 4.5 TRPC3

**Figure 5:**
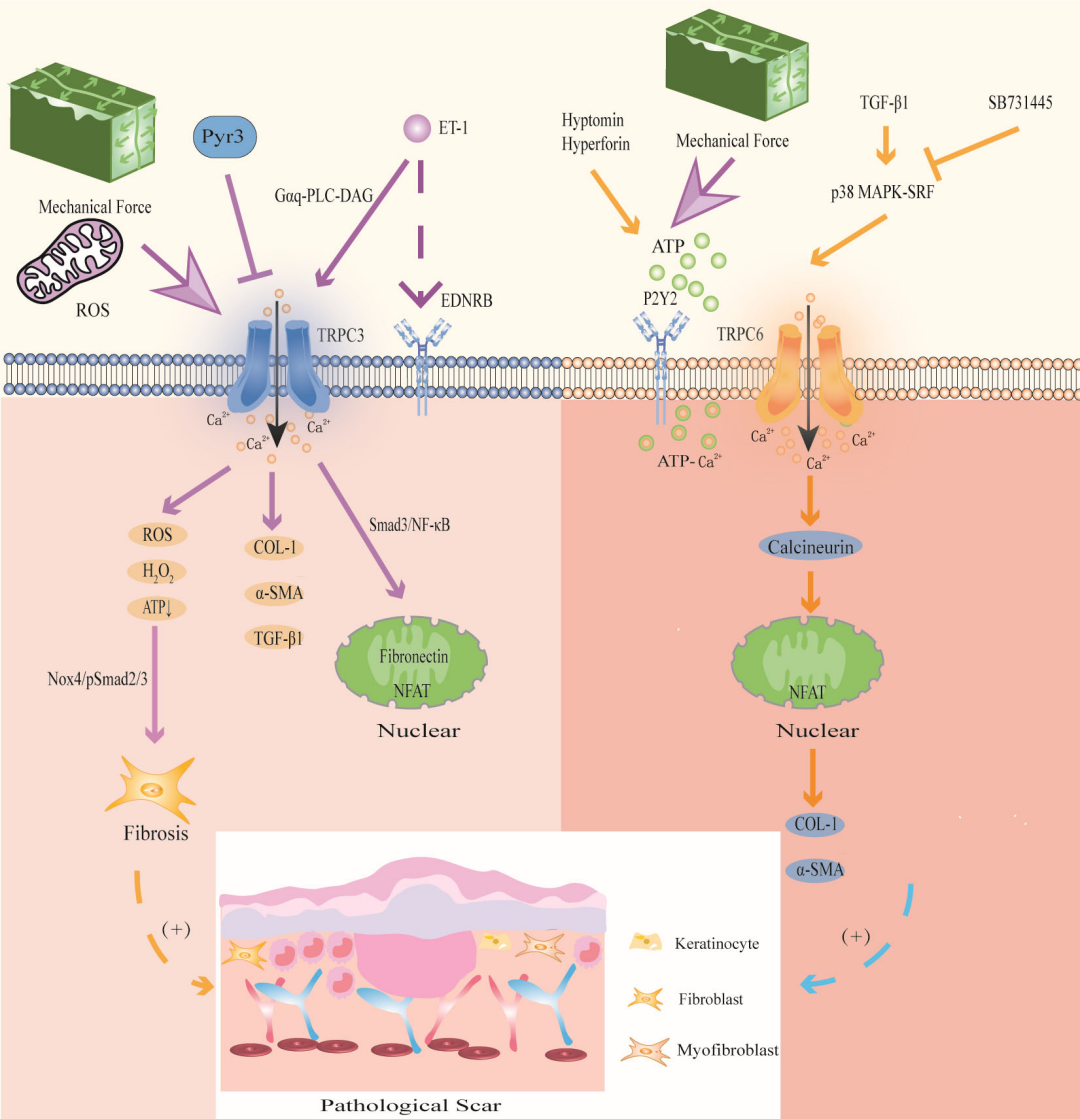
Modulation of TRPC3 and TRPC6 channels in PS. TRPC3 channel induces Ca^2+^ influx accompanied by the expression of ROS, activates Nox4/pSmad2/3 pathway, and participates in FBs differentiation; activation of TRPC3 can also directly promote the expression of COL-1, α-SMA and TGF-β1;mechanical stretching and oxidative stress activate TRPC3 channels to increase Ca^2+^ influx, and then activated Smad3/NF-κB migrates to the nucleus to induce fibrin expression and promote wound contraction; the signal exchange between ET-1 secreted by KCs and EDNRB activates TRPC3 channel and promotes the expression of the profibrotic gene NFAT. Mechanical stretching stimulates ATP release and activates TRPC6 channel upon binding to P2Y2 receptors, enhancing ATP- Ca^2+^ influx and triggering wound healing; TGF-β1 activates TRPC6 channel through p38 MAPK-SRF signaling pathway, and promotes the expression of collagen and α-SMA through TRPC6/calcineurin/NFAT signaling pathway.

TRPC3 induces mechanical stretch to promote the differentiation of FBs and KCs and regulates oxidative stress by promoting the production of ROS and H_2_O_2_ in PS (**Figure 5**).

Studies have shown that repeated mechanical stretching can promote the expression of TRPC3 in HS (33). TRPC3 promotes the expression of fibronectin through the Smad3/NF-κB pathway, thereby affecting FBs fibrosis in HS. In addition, TRPC3 protein and mRNA levels were positively correlated with VSS in HS patients (33). Further *in vitro* experiments showed that compared with that in Trpc3+/+ mice, the expression of TGF-β1, α-SMA, fibronectin and COL-1 in the granulation tissue of Trpc3-/- mice was significantly decreased. In addition, the TRPC3 inhibitor Pyr3 could significantly downregulate the expression of TGF-β1 (67). This finding indicates that mechanical stretching induces FBs fibrosis in PS by activating TRPC3 channels. In addition, the signal exchange between ET-1 secreted by KCs induced by mechanical stretch in PS and the ET-1 receptor EDNRB in FBs enhanced the expression of TRPC3 in FBs through Gαq-PLC-DAG signaling, promoting Ca^2+^ influx and the expression of the profibrotic gene NFAT (143).

TRPC3 is involved in hypoxia-induced PS. A study showed that ROS and H_2_O_2_ was significantly enhanced and ATP was significantly reduced in HS, while the TRPC3 inhibitor Pyr3 could decrease mitochondrial ROS and H_2_O_2_ and promote ATP production, thereby reducing the level of oxidative stress (67). Furthermore, the skin FBs of Trpc3-/- mice and Trpc3+/+ mice were used for detection in animal models. It was found that the levels of PDHE1α, a key subunit of the mitochondrial tricarboxylic acid cycle, and NOX4, a ROS-producing enzyme, in the skin FBs of Trpc3-/- mice, were significantly reduced compared to those of Trpc3+/+ mice (67). These results show that TGF-β1 upregulates TRPC3 expression and promotes PDHE1α phosphorylation during wound healing, resulting in increased mitochondrial ROS and H_2_O_2_ in FBs, decreased ATP production, activation of the Nox4/pSmad2/3 pathway, and thus participating in FBs differentiation in PS. We suggest that this is the mechanism by which TRPC3 promotes PS in the context of oxidative stress. Therefore, intervention with TRPC3 may be a new idea for the treatment of PS.

### 4.6 TRPC6

TRPC6 has great potential in mediating MFBs fibrosis and mechanical stretching in PS (**Figure 5**).

Genome-wide screening identified TRPC6 as an essential channel for MFBs transformation during wound healing and tissue remodeling, and TRPC6 overexpression activates MFBs differentiation (68). Further studies have shown that TGF-β1 induces TRPC6 expression through the p38 MAPK-serum response factor (SRF) signaling pathway to promote wound healing, and the p38 MAPK inhibitor SB731445 can completely block TRPC6 expression (68). Furthermore, TRPC6 is activated to induce Ca^2+^ influx and MFBs transdifferentiation through the TRPC6/calcineurin/NFAT signaling pathway, further promoting the expression of α-SMA and collagen (68). Calcineurin inhibitors can be used to treat keloids (144). The TRPC6/calcineurin/NFAT signaling pathway, which is a key signaling pathway that mediates MFBs transdifferentiation to promote wound healing, is expected to provide new intervention targets for PS and fibrotic diseases.

In addition, TRPC6 can promote wound healing through mechanical conduction. Mechanical stimulation induces HaCaT cells to release ATP, which acts as an autocrine mediator and binds to the P2Y2 receptor, activating TRPC6 channels in HaCaT cells, promoting Ca^2+^ influx, and participating in wound healing (177). Further studies have shown that the TRPC6 activators hyperforin and hypericin, which are Chinese herbal medicines that promote wound healing, can upregulate the expression of TRPC6 in HaCaT cells induced by mechanical stretching, mediate Ca^2+^ influx and enhance ATP-Ca^2+^ signaling to promote wound healing (177, 178). These studies suggest that mechanical stimulation enhances ATP-Ca^2+^ signaling by activating TRPC6 channels, which may be a potential mechanism by which TRPC6 participates in PS.

### 4.7 TRPA1

**Figure 6:**
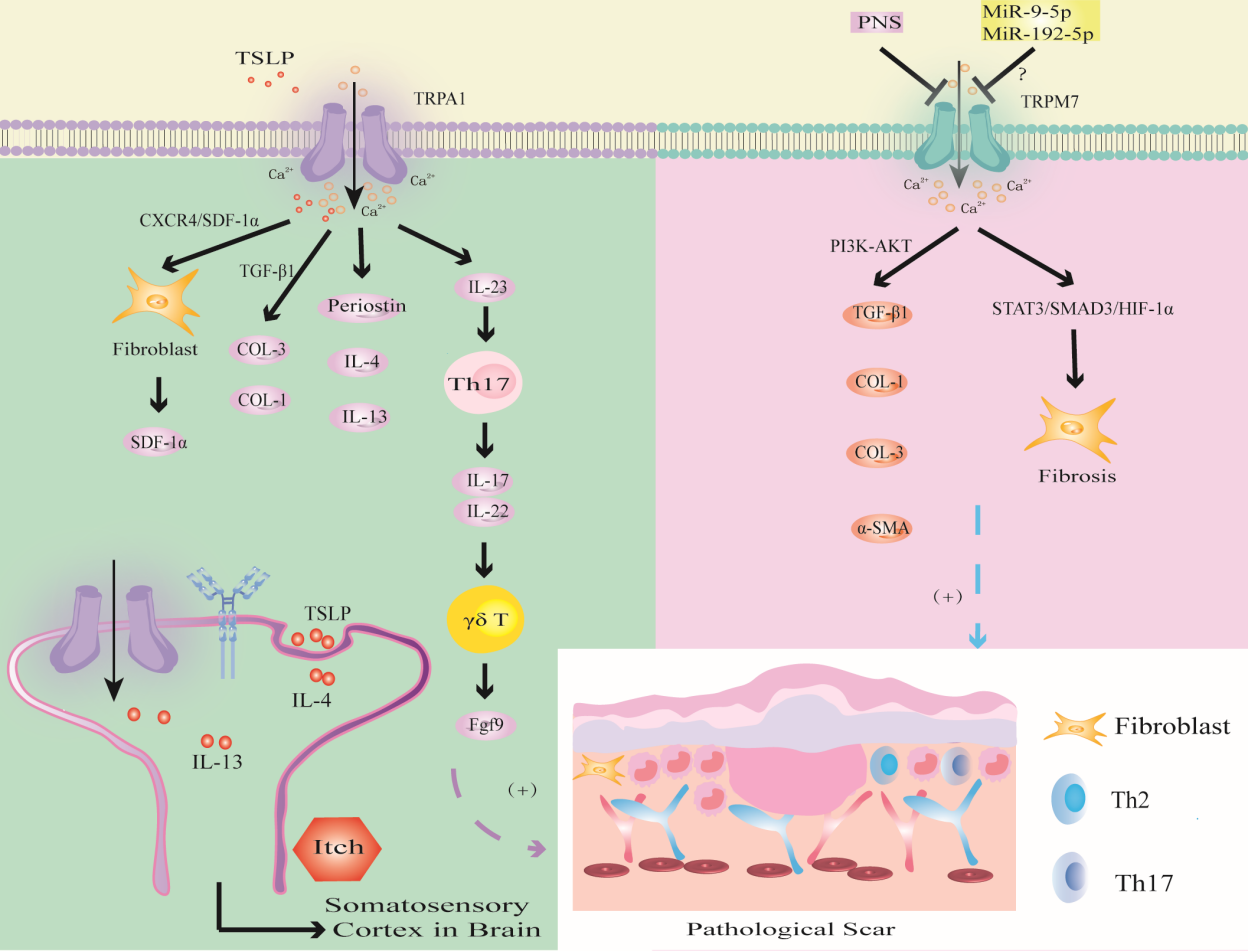
Modulation of TRPA1 and TRPM7 channels in PS. TSLP triggers TRPA1 channel to promote fibrosis through CXCR4/SDF-1 axis or promote the synthesis of COL-1 and COL-3 through TGF-β1; the activation of TRPV1 promotes the expression of Periostin, IL-4 and IL-13, which induced PS pruritus. TRPA1 activates immune cells to release inflammatory factors to promote wound healing. The activation of TRPM7 channel upregulates the expression of TGF-β1, COL-1, COL-3 and α-SMA through PI3K-AKT signaling pathway, or induces hypoxia through STAT3/SMAD3/HIF-1α signaling pathway. MiR-9-5p and miR-192-5p may inhibit PS by targeting TRPM7.

TRPA1 mainly regulates immune inflammation and fibrosis of PS. Furthermore, this channel mediates PS pruritus (**Figure 6**).

Murata found that TRPA1 deficiency inhibited the infiltration of FBs, T cells and the expression of α-SMA and COL-1 during wound healing (65). TSLP, which is a Th2 cytokine, was positively correlated with TRPA1 expression (126). Studies have shown that TSLP promotes the expression of SDF-1a in FBs through the CXCR4/SDF-1 axis and promotes the synthesis of COL-1 and COL-3 through TGF-β1, thereby inducing keloids (147). In addition, TRPA1-expressing neurons stimulate dendritic cells to produce IL-23, leading to skin inflammation, which in turn activates type 17 immune cells to produce IL-17 and IL-22 to further recruit γδT cells, which release fibroblast growth factor 9 (Fgf9) to promote wound healing (92, 145, 146). Excessive upregulation of Fgf9 may lead to PS. In addition, the absence of TRPA1-induced upregulation of TGFβ1-related signaling cascades inhibits chemical injury-induced corneal wound inflammation and fibrosis in mice (20). Thus, TRPA1-mediated inflammation and fibrosis play important roles in wound healing. Targeting TRPA1 may provide a new strategy for the treatment of PS (20).

Studies have shown that the expression of TRPA1 in the scar tissue of patients with burn scar pruritus is higher than that of patients without pruritus, especially in mast cells (141). Studies have shown that IL-4 and IL-13 can upregulate the transcription of TRPV1 and TRPA1 (126). IL-4 and IL-13 can not only mediate PS fibrosis through the TGF-β/SMAD and IL-4Rα/STAT6 signaling pathways but also directly stimulate neurons through IL-4 receptors to induce PS pruritus (87). This finding suggests that IL-4 and IL-13 secreted by Th2 cells may mediate PS pruritus through TRPV1 and TRPA1 neurons. These studies provide a new direction for improving PS pruritus.

### 4.8 TRPM7

Abnormal TRPM7 expression may be involved in PS fibrosis, oxidative stress and noncoding RNA-related gene regulation (**Figure 6**).

Zhi found that the expression of TRPM7 was upregulated and enhanced the expression of TGF-β1, COL-1, COL-3 and α-SMA through the PI3K-AKT signaling pathway in HS (179). Furthermore, Panax Noto ginseng saponins (PNSs) can protect against HS by inhibiting TRPM7 expression, cell migration and viability, and collagen deposition by downregulating the PI3K/AKT pathway, thus inducing apoptosis and cell cycle arrest. This finding indicates that TRPM7 can promote fibrosis and collagen deposition through the PI3K/AKT signaling pathway and ultimately induce PS.

Zhang found that the overexpression of TRPM7 regulated the BAX/Bcl-2 balance and antioxidant processes through the STAT3/SMAD3/HIF-1α signaling pathway, thereby promoting FBs migration and inducing wound healing (69). This led us to hypothesize that TRPM7 may accelerate PS through the STAT3/SMAD3/HIF-1α signaling pathway.

In addition, TRPM7 may be related to the regulation of epigenetic noncoding RNA-related genes in PS. MiR-9-5p mediates endothelial cell proliferation, migration and angiogenesis by targeting TRPM7 through the PI3K/AKT/autophagy pathway (180). Studies have shown that miR-9-5p can inhibit the expression of α-SMA and COL-1 in HS by targeting PPARβ, promote the proliferation of FBs and accelerate apoptosis (181). Therefore, we hypothesize that miR-9-5p targets PPARβ to activate TRPM7 channel through the PI3K/AKT pathway, inhibit FBs fibrosis and collagen deposition, which may be an important target for PS. In addition, studies have shown that expression of the tumor suppressor miR-192-5p can be reversed by TRPM7 overexpression, thereby promoting the proliferation, migration and invasion of cervical cancer cells (182). However, miR-192-5p derived from adipose tissue-derived mesenchymal stem cell-derived exosomes (ADSC-Exos) inhibits HS by targeting IL-17 to regulate the Smad pathway (183). Therefore, inhibiting miR-9-5p to promote the expression of TRPM7 may aggravate PS fibrosis by activating the IL-17/Smad pathway. In-depth study of TRP channels may become an important research direction to determine the mechanism of PS in the context of epigenetics.

We now acknowledge that while few studies have reported on the involvement of other TRP channels in the mechanism of PS, the similarity of the structure and function of the homologous TRP channel family leads us to speculate that other TRP channels may also be related to the mechanism of PS, which needs further research and exploration in the future. In recent years, studies have found that the expression of TRPC1 is associated with renal fibrosis. CircRNA_010383 colocalized with miR-135a, and that overexpression of circRNA_010383 increased the level of TRPC1, which is a target protein of miR-135a. Additionally, the overexpression of circRNA_010383 inhibited the high glucose-induced accumulation of ECM (184). TRPC1 may have a potential relationship with the fibrosis of PS, but further research is needed to confirm this. Additionally, the study mentions that TRPM2 ablation has been found to significantly reduce renal interstitial fibrosis by decreasing TGF-β1 levels. This reduction in TGF-β1 is accompanied by a decrease in α-SMA, connective tissue growth factor (CTGF), fibronectin, and COL-1. The study also suggests that TRPM2 ablation may protect against renal fibrosis and inflammation by impeding JNK activation regulated by TGF-β1 (185). TRPM2 may be involved in inflammation and fibrosis of PS. Additionally, TRPML channels have important functional activities in immune cells such as macrophages, dendritic cells, neutrophils, NK cells, and B lymphocytes, which may provide important ideas for exploring the mechanism of TRPML in the inflammation and immunity of PS (186).In addition, TRPP1-like proteins may form mechanosensors in primary cilia. The phosphorylation of the COOH-terminus of TRPP1 may recruit TRPP2 to the cell membrane, which can promote Id2 to enter the nucleus and induce renal cell proliferation (187). The study suggests that Id2, as a key regulator of cell proliferation and differentiation, may provide a research direction for future studies on the mechanism of TRPP channel and PS.

## 5 Discussion and conclusion

In recent years, it has been found that a variety of TRP channels are involved in the development of PS, especially in immune inflammation and fibrosis, which has great research and clinical values. In this review, we introduced the specific physiological and pathological functions of TRP channels and focused on their important roles in mediating PS immune inflammation, fibrosis, mechanical conduction, epigenetics, oxidative stress and pruritus. TRP channels promote tissue fibrosis and re-epithelialization by regulating the proliferation, migration, phagocytosis and cytokine release of immune cells or by directly activating FBs and KCs to promote the deposition of ECM and mediate the development of PS; mechanical stimulation which directly promotes fibrosis by activating TRP channels or recruiting immune cells to participate in fibrosis; noncoding RNA which regulates FBs fibrosis and collagen deposition by activating TRP channels; and the activation of TRP channels that can promote the release of oxidative stress-related factors such as ROS and H2O2, which in turn induce PS. Based on the latest reports, this review summarized the most novel concepts for the therapeutic drugs of PS from targeting TRP channels as follows: EVs are paracrine molecules that regulate cell signal transduction in the corneal scar and fibrosis, and studying how EVs participate in the development of PS through TRP channels may provide a promising treatment (167); Mechanical forces stimulate some TRP receptors on skin sensory fibers to release neuropeptides including SP and CGRP, which may provide direction for TRP-mediated PS neurogenic inflammation mechanism (122); and IL-31 mediates pruritus through TRPV1 and TRPA1 channels, indicating a new therapeutic target for the treatment of PS pruritus (127, 128). In addition, most of the current research on PS focuses on immunity. Immune signals enhance epithelial cell proliferation, differentiation and migration to accelerate skin repair (188), which suggests that targeting immune-epithelial communication to promote repair or reduce inflammation can be a new strategy for PS treatment. Therefore, studying the important role of TRP channels on account of the immune perspective may prompt novel targets for improved treatment of PS.

Although many studies have been carried out on immune inflammation in PS, the role of TRP channels in the regulation and treatment of PS is still in its infancy, mainly because it is a relatively new field involving wound healing and PS mechanisms, and many problems require further study. First, in response to different intensities of mechanical stretching, some mechanoreceptors in the TRP family promote wound healing. Excessive activated TRP in the wound site may lead to PS. Therefore, identifying the most suitable mechanical force triggering wound healing is particularly important to avoid the occurrence of PS. Second, the effects of various TRP channels on PS are different. For instance, the mechanism of TRP channels in the inflammatory, proliferative and remodeling stages of PS, TRP needs to be further explored. Finally, although several TRP agonists or antagonists have been developed for the treatment of PS, such as SKF96365 or tranilast, they have infrequently used in clinical practice due to the problems of drug permeability, side effects, and poor efficacy (189). Therefore, there is an urgent need for a comprehensive summary and in-depth evaluation of TRP channels to provide novel directions for identifying more selective and efficacious drug target to treat PS. This paper summarized the latest progress in understanding the role of TRP channels in PS, and the role of other TRP channels in PS needs to be further examined.

In the future, as the mechanisms of different TRP channels and their interactions with cytokines and growth factors become increasingly clear, TRP channels are expected to have further breakthroughs in the prevention and treatment of PS. In this article, we summarized the important roles of TRP channels in the pathogenesis of PS and proposed that drug development targeting TRP channels will be a critical topic for PS. Therefore, we hope to provide potential pharmacological targets and directions for future deeper understanding of PS and the evolution of new drugs by summarizing the mechanism of TRP channels in PS.

## Author contributions

YPZ conceived and wrote the article. QH, YFZ, and LG collected the data. WW was responsible for the idea. HZ was responsible for conceptualization and funding. XH was responsible for project administration and funding. QL were responsible for the idea, funding, and paper revision. All authors contributed to the article and approved the submitted version.
